# Correction: Evaporation-driven liquid flow in sessile droplets

**DOI:** 10.1039/d3sm90023a

**Published:** 2023-02-23

**Authors:** Hanneke Gelderblom, Christian Diddens, Alvaro Marin

**Affiliations:** a Department of Applied Physics and Institute for Complex Molecular Systems, Eindhoven University of Technology The Netherlands h.gelderblom@tue.nl; b Physics of Fluids, University of Twente The Netherlands c.diddens@utwente.nl a.marin@utwente.nl; c J.M. Burgers Center for Fluid Dynamics The Netherlands

## Abstract

Correction for ‘Evaporation-driven liquid flow in sessile droplets’ by Hanneke Gelderblom *et al.*, *Soft Matter*, 2022, **18**, 8535–8553, https://doi.org/10.1039/D2SM00931E.

In the published article, in Section 3.1 “Thermal Marangoni flow”, there is a typo in the estimation of Δ*T* proposed,† it should read Δ*T* = *H*_v_/*C*_p_. While this expression is dimensionally correct and gives rise to the definition of the Marangoni number in eqn (23), it does not give realistic values for evaporation-driven cooling. A better approximation would be:
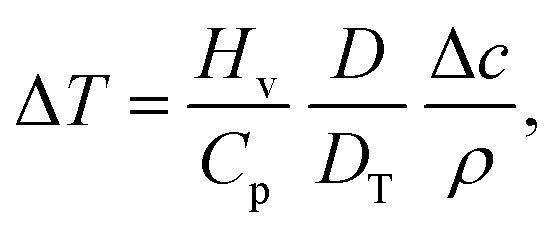
where *H*_v_ is the latent heat of vaporization, *C*_p_ is the specific heat capacity at constant pressure (all defined in Section 3.1), *D* is the diffusion coefficient of the vapor in air, *ρ* is the liquid density, Δ*c* is the difference between the saturated vapor concentration at the interface and in the far field (all defined in Section 2.1) and *D*_T_ is the thermal diffusivity of the liquid phase.

The results and conclusions presented in the published article are unaffected.

† The authors would like to thank Jafar Farhadi for pointing out the typo.

The Royal Society of Chemistry apologises for these errors and any consequent inconvenience to authors and readers.

## Supplementary Material

